# Supplementary material for: The Usage of Antibiotics by COVID-19 Patients with Comorbidities: The Risk of Increased Antimicrobial Resistance
Source: Antibiotics (Basel). 2021 Dec 29;11(1):35. doi: 10.3390/antibiotics11010035 (PMC8772884; doi:10.3390/antibiotics11010035)
Supplement: Supplementary file 1 [file antibiotics-11-00035-s001.zip › antibiotics-1522524-supplementary.pdf]

Supplementary Materials

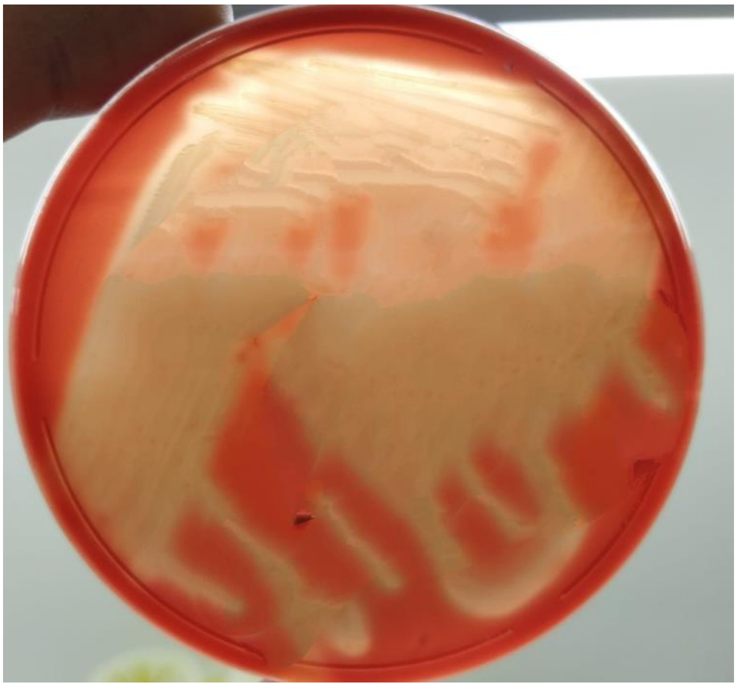

Figure S1. Clear beta Hemolysis by *Streptococcus*.

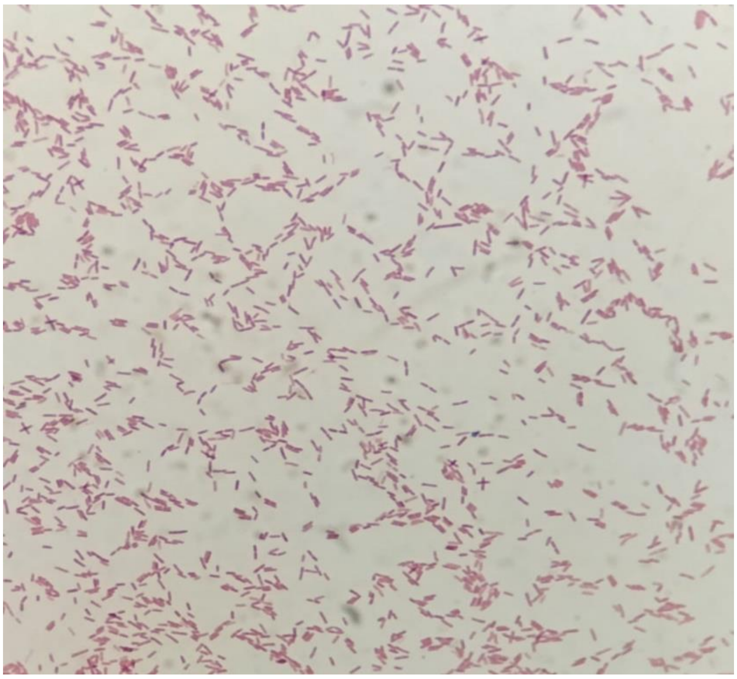

Figure S2. Gram stain showing Gram-Negative bacteria.

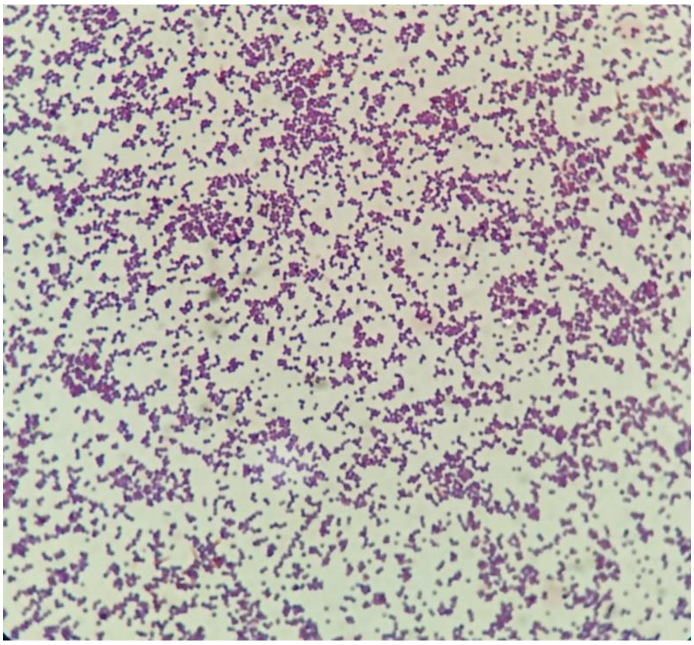

Figure S3. Gram Stain showing Gram-Positive Bacteria.

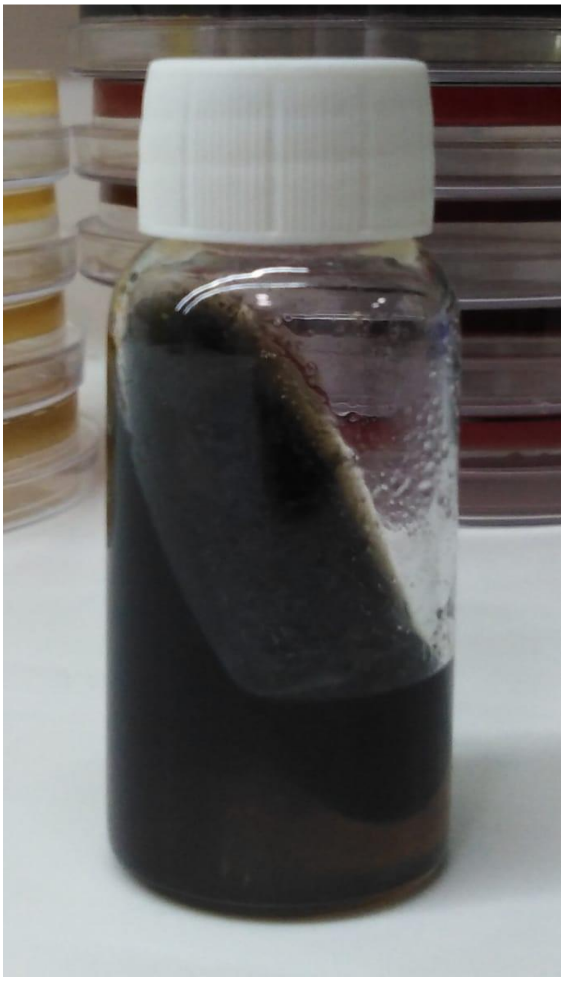

Figure S4. A positive Bile Asculine Reaction (blacking of media).

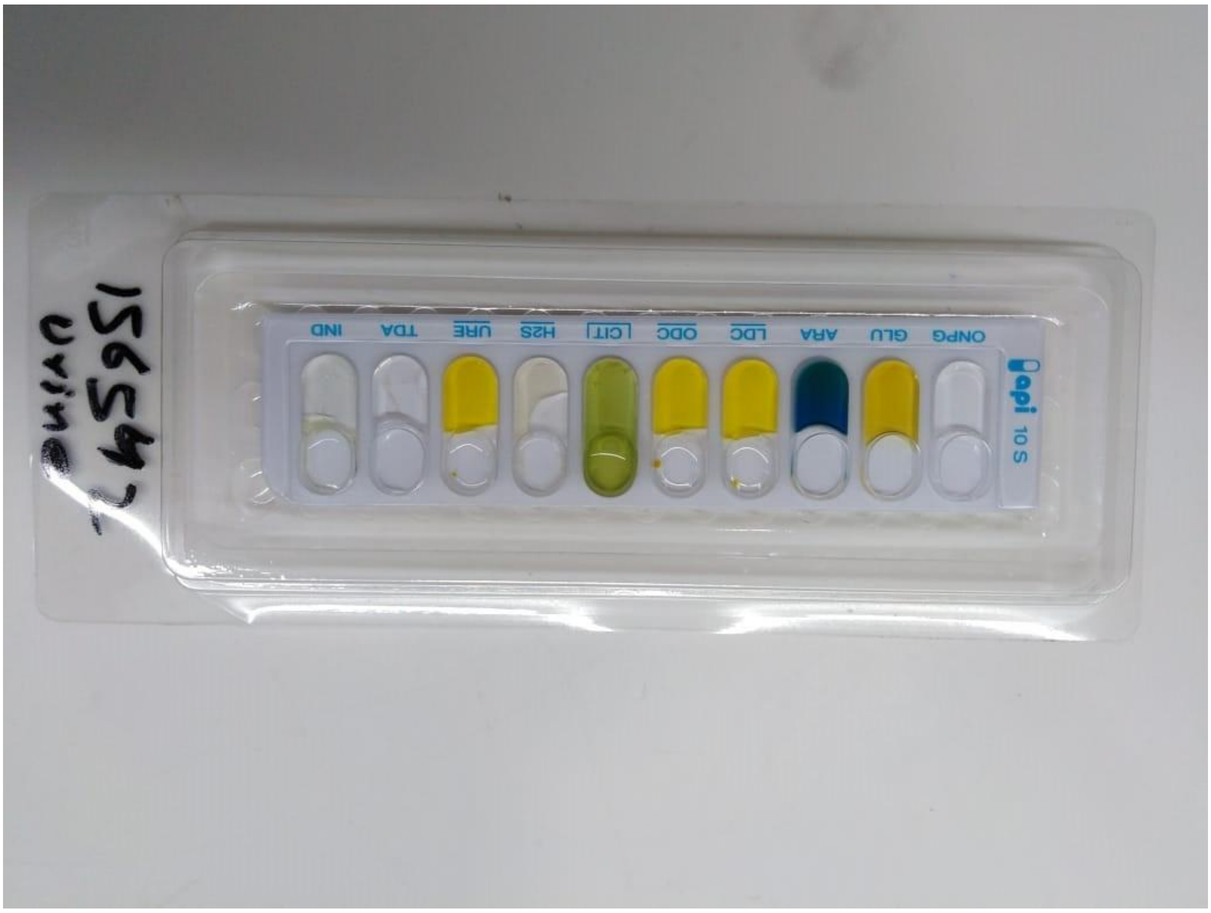

Figure S5. Result of *E. coli* on API.
